# In Vitro and In Vivo Evaluation of Oral Controlled Release Formulation of BCS Class I Drug Using Polymer Matrix System

**DOI:** 10.3390/ph14090929

**Published:** 2021-09-16

**Authors:** Mosab Arafat, Muhammad Sarfraz, Mohammad F. Bostanudin, Anna Esmaeil, Aisha Salam, Salahdein AbuRuz

**Affiliations:** 1 College of Pharmacy, Al Ain University, Al Ain P.O. Box 64141, United Arab Emirates; mosab.arafat@aau.ac.ae (M.A.); muhammad.sarfraz@aau.ac.ae (M.S.); mohammad.bostanudin@aau.ac.ae (M.F.B.); annaesmaeel723@gmail.com (A.E.); aisha.salam@aau.ac.ae (A.S.); 2 Department of Pharmacology and Therapeutics, College of Medicine and Health Sciences, United Arab Emirates University, Al Ain P.O. Box 17666, United Arab Emirates; 3 Department of Biopharmaceutics and Clinical Pharmacy, School of Pharmacy, The University of Jordan, Amman 11942, Jordan

**Keywords:** diltiazem hydrochloride, poloxamer-188, matrix system, polymer, controlled release

## Abstract

Diltiazem hydrochloride is a calcium channel blocker, which belongs to the family of benzothiazepines. It is commonly used to treat hypertension and atrial fibrillation. Even though the drug has high solubility, its high permeability and rapid metabolism in the liver can limit the bioavailability and increase the dose frequencies for up to four times per day. This study focused on a polymer matrix system not only to control the drug release but also to prolong the duration of bioavailability. The polymer matrices were prepared using different ratios of poloxamer-188, hydroxypropyl methylcellulose, and stearyl alcohol. In vitro and in vivo assessments took place using 24 rabbits and the results were compared to commercially available product Tildiem^®^ (60 mg tablet) as reference. Overall, the rate of drug release was sustained with the gradual increase of poloxamer-188 incorporated with hydroxypropyl methylcellulose and stearyl alcohol in the matrix system, achieving a maximum release period of 10 h. The oral bioavailability and pharmacokinetic parameters of diltiazem hydrochloride incorporated in polymer matrix system were similar to commercial reference Tildiem^®^. In conclusion, the combination of polymers can have a substantial effect on controlling and prolonging the drug release pattern. The outcomes showed that poloxamer-188 combined with hydroxypropyl methylcellulose and stearyl alcohol is a powerful matrix system for controlling release of diltiazem hydrochloride.

## 1. Introduction

Diltiazem hydrochloride (DLZ), a benzothiazepine, acts as a calcium channel blocker [1], which makes it useful in the treatment of many diseases, including angina, supraventricular tachyarrhythmia, and hypertension [2]. According to the bio-pharmaceutics classification system (BCS), it belongs to class I drugs based on its high water solubility [3] and high permeability [4]. Despite high absorption after oral administration, the bioavailability of DLZ was reported to be poor because of its extensive liver metabolism, with only a maximum of 40% of drug molecules reaching the blood systemic circulation system [5]. Therefore, it has a short half-life of about 3–5 h [6]. Because of these facts, DLZ was chosen as candidate drug for developing a controlled release polymer matrix system that can overcome such shortcomings. Pharmaceutical formulations typically intend to deliver therapeutic agents in a predictable and reproducible manner over an extended period of time after single dose administration [7]. Therefore, the ultimate goal is to tailor the components in a dosage form to such an extent that it leads to foreseeable alterations in release profiles. Conventional dosage forms, however, often fail to define drug absorption into the body [8]. To fulfill this criterion, the design process of a controlled release system should also include appropriate characterization of drug permeation through the relevant biological membranes and the first pass metabolic effect prior to entering the blood circulation [8]. Controlled release systems not only promote drug efficacy, bioavailability, and pharmacokinetics, but also minimize side effects by delivering the proper amount of drug molecules to the target sites at wanted rates based on zero-order elimination kinetics [9].

To date, oral solid dosage forms are the most studied delivery systems in the pharmaceutical sciences [10]. The countless number of existing and novel drugs, however, has led to continuous efforts to establish new formulas, technologies, and devices to regulate their release profiles [10]. The majority of studies have been focused on enhancing drug bioavailability and reducing dosing frequencies [11,12]. This can be achieved by several pharmaceutical approaches, including, for example, coating systems [13], osmotic pressure systems [14], and matrices systems [15]. They can be made of polymers [16,17], lipids [18], chitosan [19], starch [20], cellulose [21], polyester amide [22], polycarbonates [23], or hydroxypropyl methylcellulose (HPMC) [24]. 

Matrix Systems are the earliest and most frequently used method to modify release profiles of drugs [18]. Here, the drug is homogeneously dissolved or dispersed in the polymeric matrix [18]. In particular, slowly dissolving or erodible matrices provide a simple way of retarding the release rate of drugs with rapid dissolution. In a matrix system, the drug molecules form a homogenous mixture with the rate controlling material being amorphous, crystalline or, rarely, a molecular dispersion [25]. The drug can be released by diffusion and/or dissolution [26]. Hydrophilic polymers are the most common matrix systems for oral delivery dosage forms [18] because of cost-effectiveness, wide regulatory approvals, reduced toxic effect, and they provide approachable drug release profiles [24]. Matrix systems are applicable for controlling the release of many active molecules [27]. They can be used for water-soluble and insoluble drug molecules [28,29], as hydrophilic or hydrophobic polymeric materials can be selected accordingly [30,31]. Amphiphilic polymers are a promising alternative to achieve controlled release [32], as their dual character can broaden applications for both water-soluble and insoluble drug molecules [33]. 

Poloxamers are one of the recently used triblock polymers with an amphiphilic nature, in which the central polypropylene oxide forms the hydrophobic part and the two outer polyethylene oxide blocks acts as the hydrophilic portion [34,35]. Its amphiphilic character can be varied by adjusting the number of central and side chains in the copolymer [36]. Being an amphiphile, poloxamers can self-assemble in an aqueous medium enhancing solubility and dissolution of poorly water-soluble drugs [37,38]. The main release mechanism of the active molecules can be either by diffusion or erosion [39]. 

Various poloxamers types are available on the market, including poloxamer 188 (P-188), poloxamer 184 (P-184), poloxamer 401 (P-401), poloxamer 108 (P-108), poloxamer 402 (P-402), poloxamer 408 (P-408), and poloxamer 407 (P-407) [40]. The application of P-188 to develop controlled release systems has been reported previously, for example, for tetramethylpyrazine [41], sildenafil citrate [42], and indomethacin [43]. However, to the best of our knowledge, DLZ-modified release preparation using different dosage forms and materials have been reported [44,45], but no study has used P-188 in a controlled release matrix system for DLZ. 

In vitro dissolution experiments are a powerful tool in the development of oral controlled release preparations. Various variables affecting the drug release can be investigated, particularly during the developmental stage, providing the basis for the final formulation with the desired in vitro drug release parameters. While in vitro evaluations can provide information on product uniformity and the effect of changes in the formulation itself, they cannot necessarily predict the in vivo situation [32]. In vivo assessment is particularly required to demonstrate the clinical efficacy of a dosage form. This is especially vital for prolonged release medication, where the programmed rate of release and extended period of absorption are more critical [31]. Thus, in vivo studies are essential in order to evaluate oral bioavailability and to assess the pharmacokinetics (PK) parameters.

The main aim of this study was to develop and prepare a controlled release dosage form for DLZ, a BCS Class I drug. A mixture of P-188 with HPMC and stearyl alcohol (STA) formed the polymer matrix system. After evaluation of in vitro drug release, in vivo investigations of the polymer matrix system were performed using 24 rabbits to evaluate the oral bioavailability, pharmacokinetics (PK) of the drug and the significance of the in vitro data. Commercial product Tildiem^®^ (60 mg tablet) was used as reference in each experiment. 

## 2. Results

### 2.1. Drug Release and In Vitro Evaluation 

**Figure 1:**
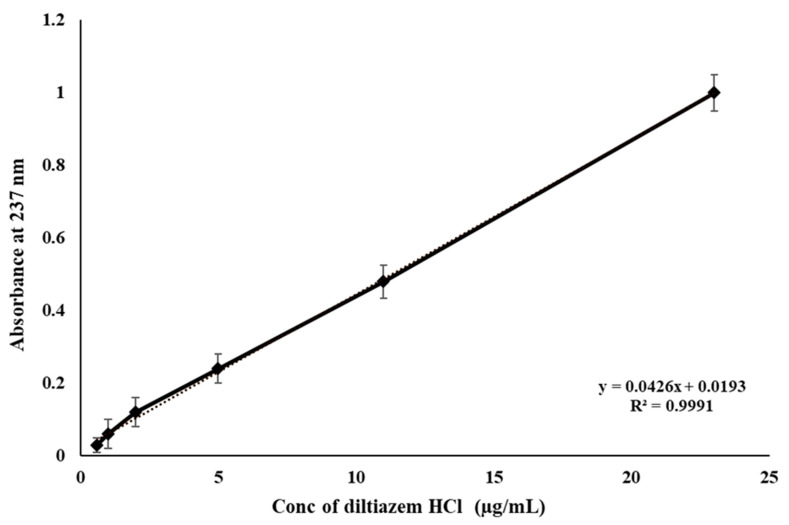
DLZ calibration curve in distilled water at 37 °C, (*n* = 6), values are means ± S.D. (*n* = 6).

The calibration curve of DLZ was prepared in replicate of six (*n* = 6) to obtain the best-fit line and regression equation. It was performed by using concentrations of 0.6 to 23 µg/mL of the drug in distilled water. Drug concentrations were measured using UV spectrophotometry and absorbance was detected at 237 nm wavelength. The average regression coefficient (r^2^) of DLZ showed an ideal linear regression line with r^2^ value of 0.9991 (Figure 1). In addition, the drug content in the polymeric matrices were determined and found to be 60 ± 3.1 mg. 

**Figure 2:**
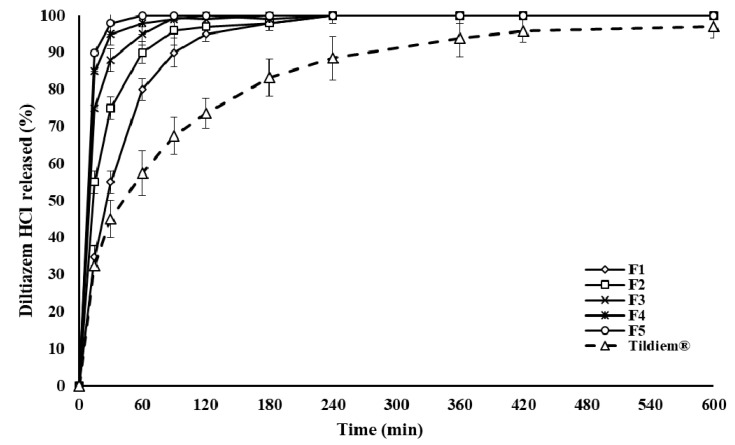
The rate of DLZ-controlled release from polymer matrices of various drug to polymer percentages and Tildiem^®^ over 600 min, using distilled water at 37 °C, values are means ± S.D. (*n* = 6).

**Figure 3:**
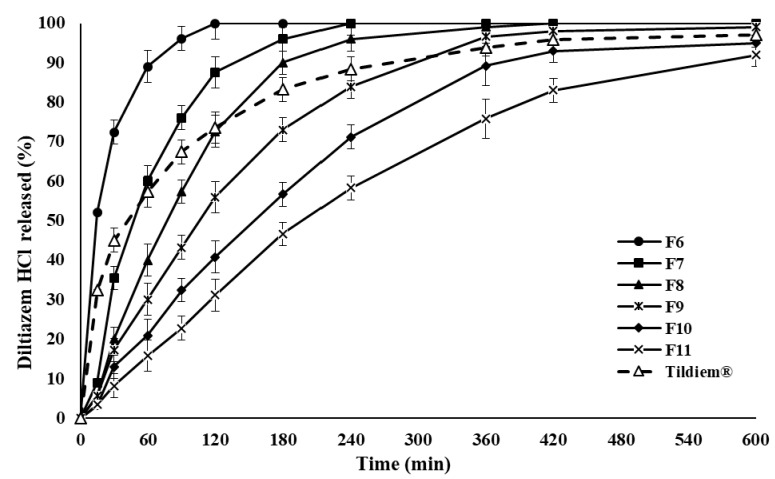
The rate of DLZ controlled release from polymer matrices containing constant amount of drug and various polymer mixture percentages and Tildiem^®^ over 600 min, using distilled water at 37 °C, values are means ± S.D. (*n* = 6).

The dissolution results of Tildiem^®^ (60 mg tablet), the commercial reference, is shown in Figure 2. The release rate of the drug molecules was in a controlled manner over seven h. Additionally, Figure 2 illustrates the drug release rates from the first series of formulations (F1–F5). The percentages of incorporated drug into the polymeric matrix to P-188 are presented in Table 1. A significant drop in the release rate occurred with an increasing percentage of P-188 in the polymer matrix. The dissolution of DLZ was in the order Tildiem^®^ > F1 > F2 > F3 > F4 > F5. It is evident from the results that the release rate of the drug can be decreased in a controlled manner by gradually rising the content of P-188. However, this was only applicable for up to four h. Figure 3 shows further reduction in the drug release profiles of DLZ for up to 10 h. Here, in the second series of formulation, only 20% of drugs are incorporated into a matrix system composed of various percentages of P-188: HPMC: STA mixture (F6–F11) as listed in Table 2. The drug release rate reduced in the order F6 > F7 > F8 > F9 > F10 > F11. A remarkable further reduction in the drug release rate was achieved as the percentage of STA was gradually increased. The maximum in delay was obtained at 15% incorporation of STA, substituted for the 50% portion of HPMC in the preparations.

**Figure 4:**
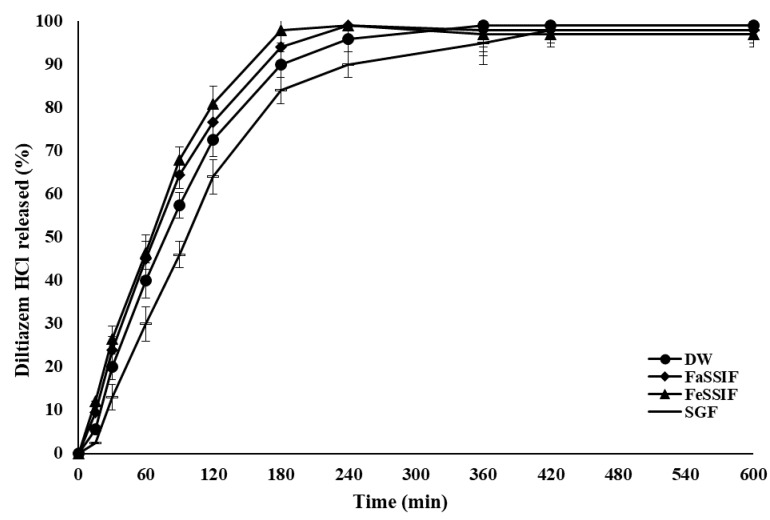
The rate of DLZ controlled release from polymer matrices of test formulation (F8) over 600 min, using different simulated physiological media namely: FaSSIF, FeSSIF and SGF in comparison to distilled water (DW) at 37 °C, values are means ± S.D. (*n* = 6).

Figure 4 shows the drug release rates from test formulation (F8) incubated in various simulated physiological media, namely: fast simulated small intestine fluid (FaSSIF), fed simulated small intestine fluid (FeSSIF), and simulated gastric fluid (SGF). The rates of drug release from test formulation incubated in three different physiological simulated media were comparable and only slight differences were occurred. The results indicated that the rate of DLZ release from the polymer matrices of test formulation were obviously independent of the dissolution media. 

### 2.2. Mechanism of Drug Release from Polymer Matrix 

Table 3 shows the *n* values of DLZ for selective formulations (F7-F10) with corresponding r^2^ values, where r^2^ is the coefficient of determination of the drug release mechanism of the respective preparations. Hixson Crowell and zero order kinetic release appeared to be the best fit model to explain the progress of drug release.

### 2.3. Thermal Profiles of DLZ in P-188 Polymer Matrix

**Figure 5:**
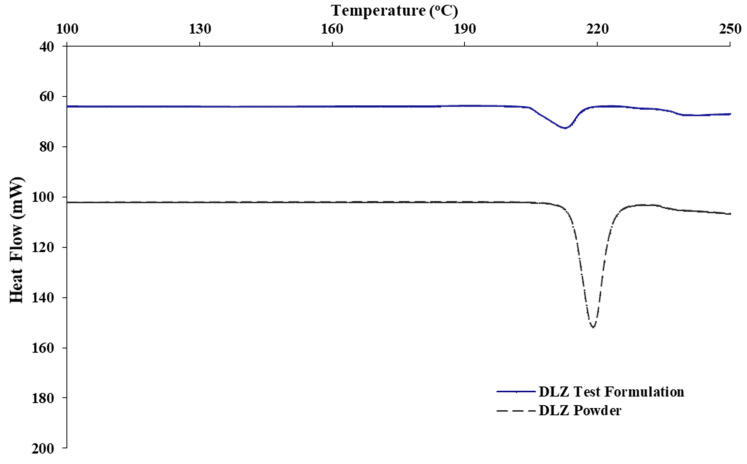
DSC Thermal profile of DLZ dispersion in test formulation and in pure crystalline powder.

Figure 5 illustrates the thermal profiles of DLZ from test formulation (F8) compared to pure drug powder. Upon incorporation of drug into the polymer matrix, it can be observed that the melting peak shifted from 220 to 211 °C and a reduction in the enthalpy of fusion (Δ*H*_f_) occurred from 125 ± 7.4 to 79.6 ± 3.1 J/g.

### 2.4. HPLC

**Figure 6:**
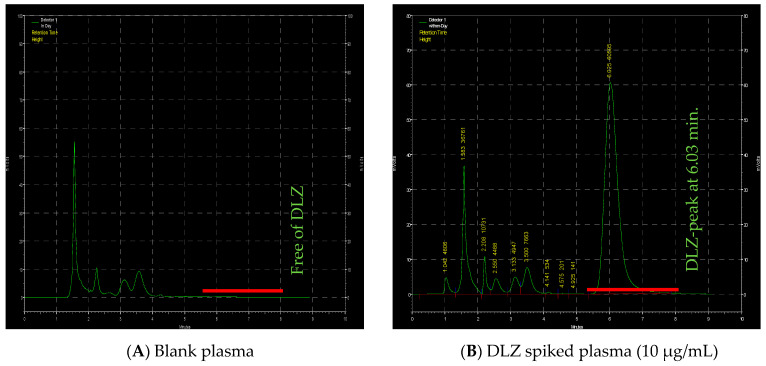
Chromatograms of DLZ obtained from (**A**) plasma free of DLZ and (**B**) plasma spiked with10 µg/mL DLZ (Reprinted from ref. [46]).

The chromatogram showed a well-resolved DLZ peak with a retention time of 6.03 min in plasma spiked with 10 µg/mL DLZ (Figure 6). A duplicate of six plasma spiked with DLZ batches were tested over 10 min run time. DLZ was found to be stable in plasma at room temperature, with no noticeable changes in DLZ plasma concentrations. Calibration curves were prepared over a concentration range of 0.25–20 µg/mL. Good linearity was obtained for DLZ in both plasma (r^2^ 0.9998 ± 0.0007) and in water (r^2^ 0.9997 ± 0.0003). The selected concentration range showed excellent absolute recovery (Table 4). Intra-day and inter-day accuracies were 95.1–106.7% and 94.1–103.4%, respectively, with precision of (CV) ≤ 7.5%. The limit of detection (LOD) was 0.125 µg/mL and the limit of quantification (LOQ) was 0.25 µg/mL. At this lowest concentration, intra-day and inter-day precision were 5.2% and 3.8%, respectively. The obtained values were in good agreement with previous reports data [46]. 

### 2.5. In Vivo Assessment of Drug

**Figure 7:**
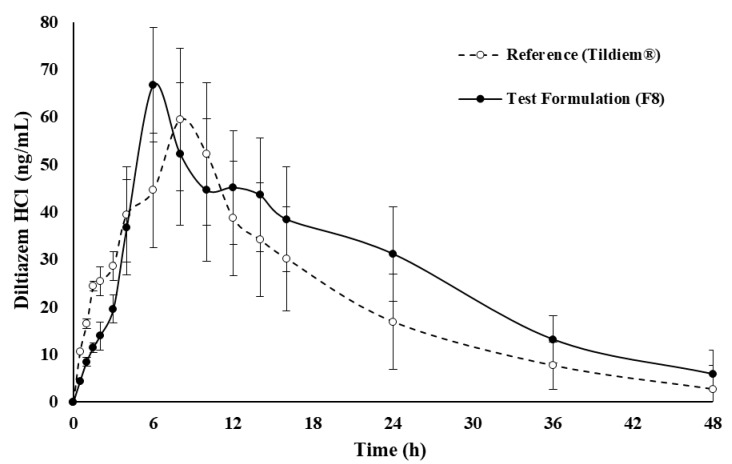
Plasma concentration of DLZ after oral administration of the commercial reference Tildiem^®^ (60 mg tablet) and test formulation (F8), each point represents the mean ± SD, *n* = 12.

Figure 7 shows the in vivo profile of DLZ in rabbit plasma, following oral administration of test formulation (F8) and commercial reference Tildiem^®^ (60 mg tablet). Both preparations showed that the plasma concentration profiles were slow and sustained for up to 48 h. Peak concentration was achieved at approximately 6–8 h after administration, followed by drug elimination over 48 h. The higher plasma concentration of test formulation (F8) indicates a comparably faster rate of absorption. The plasma concentration area under the curves of the two formulations were almost similar to each other, with slight differences attributable to body weight variation among animals. Table 5 shows respective PK values of both preparations. No statistical differences (*p* > 0.05) were observed between all PK values except for minor differences in T_max_ and C_max_.

## 3. Discussion

In the first series of formulations (F1–F5), P-188 was used alone as a matrix system in order to evaluate the impact of polymer concentration on the dissolution rate of drug. A limited controlled release of DLZ was achieved from the preliminary test formulation for up to four h compared to seven h observed for commercial reference Tildiem^®^. In the second series of formulations (F6–F11), however, the combination of P-188, HPMC and STA as polymer matrix was able to sustain the release of the drug for up to 10 h. Multiple factors can have an influence on the drug release profile from polymer matrices. Examples are the, polymer molecular weight, the degradation rate, ratio, types, binding affinity, melting point, density, and HLB values [47]. Therefore, we assumed that the controlled release of DLZ from the polymer matrix was the consequence of several aspects. First, the amphiphilic nature of P-188 most likely plays an essential role in controlling the drug release rate. Drug particles immersed in the hydrophilic outer layer might diffuse gradually into the aqueous dissolution medium, but a certain amount of the drug presumably remains in the hydrophobic inner core of the self-assembled polymer [48]. Second, the presence of HPMC could result in a relatively thicker gel layer formation on the surface of the matrix system, causing the polymer barrier to be relaxed and loosened [49,50]. Third, the addition of STA showed a significant contribution towards prolonging the drug release. The inclusion of a component of comparably wax-lipid based materials with a low melting point and density most likely prevented rapid DLZ release from the matrix system, wherein density alteration is known to have significant influence on drug release rates [51].

Therefore, the overall release mechanism of the polymer matrix system can be described as follows. The release rate of DLZ increased inversely proportional to the amount of polymer mixtures incorporated as has often been observed in the literature [52]. Upon contact with water, the polymer hydrated and the formation of a gel layer on the surface of the matrices took place, strongly influencing the mechanism of DLZ release from the test formulation [53]. Here, the gel layer might control the drug diffusion through the matrices by acting as a permeable barrier. The presence of P-188, however, increases the viscosity of the gel layer, which can alter gel networking [54]. As a result, the diffusion rate of DLZ throughout the gel layer of the matrices occurs in a controlled manner. 

DSC thermogram of F8 formulation showed a reduced melting point at 211 °C compared to 220 °C for pure drug powder. The slightly lower heat stability was attributed to certain solvation of the drug being incorporated into the polymer matrix [55]. The decrease in the area under the melting peak and the enthalpy of fusion from 125 ± 7.4 to 79.6 ± 3.1 J/g suggested a drop in drug crystallinity. However, a significant percentage of DLZ did not dissolve in the polymer matrices and was retained in crystalline form in the formulation.

For in vivo studies, the test formulation showed a slow plasma concentration profile available for up to 48 h, which was comparable to commercial reference Tildiem^®^. However, its higher plasma concentration was an indication of a faster rate of absorption compared to the reference. It might also be due to an inter-subject variation in plasma levels for both preparations, which can be attributed to differences in drug disposition and weight among animals [56]. The slight differences in C_max_ and T_max_ values for both drug dosage forms supported an enhanced absorption rate of the test formulation relative to the commercial reference. Overall, it can be concluded that the polymer matrix system is comparable to the solid dosage form on the market in terms of rate and extent of absorption. 

In addition, the pharmacokinetics parameters obtained in this study were in good agreement with values presented in the literature [52,53]. The parameters T_max_ and AUC_0-∞_ are correlated to the rate and extent of drug absorption, respectively, while C_max_ is associated with both processes [54]. In a comparative bioavailability study, the value of AUC_0-∞_ is often considered as the most important parameter, as the extent of absorption is a key characteristic of drug formulations. The other two parameters, namely T_max_ and C_max_, are of particular interest in describing the plasma level profile, as they are connected to the controlled release profile of the drug dosage forms. Here, the reference showed a higher mean T_max_ compared to the test formulation. This would be an indication for a relative higher absorption rate of commercial Tildiem^®^. Similarly, the values of T_max_ obtained for both preparations were of no significant difference (*p* > 0.05). Moreover, no statistical difference (*p >* 0.05) could be detected between log-transformed AUC_0–∞_ values and log-transformed C_max_ values of the two preparations.

## 4. Material and Methods

### 4.1. Chemicals

Poloxamer-188 was purchased from Merck (Darmstadt, Germany). Diltiazem hydrochloride (DLZ), stearyl alcohol (STA), and hydroxypropyl methylcellulose (HPMC) were obtained from Thermo Fisher Scientific (Loughborough, UK). Tildiem^®^ (60 mg tablets) were ordered from Al Ain Pharmacy (Al Ain, UAE). Lecithin was acquired from Lipoid GmbH (Ludwigshafen, Germany). Sodium taurocholate (NaTC) and pepsin were obtained from Sigma Aldrich (St. Louis, MO, USA). Acetonitrile, 2−propanol, n-hexane, isopropyl alcohol, trimethylamine, ammonium dihydrogen phosphate, phosphoric acid, hydrochloric acid, glacial acetic acid, and sodium hydroxide were acquired from Merck (Darmstadt, Germany). All solvents used were of HPLC grade. 

### 4.2. Preparation of Controlled Release Polymer Matrix Containing DLZ

Two series of polymer matrix formulations were prepared. For series 1, the polymer matrix containing DLZ in P-188 was made as follows: P-188 was melted in a water bath at 50 °C and allowed to stir for 20 min at 200 rpm. DLZ was added, and the mixture was stirred for a further 20 min. Size 00 hard gelatin capsules were used for the obtained liquefied dispersion. All preparations were kept at a room temperature until the preparation solidified, and afterwards stored in the fridge at 8 °C. 

For series 2, the polymer matrix containing DLZ in a combination of P-188, HPMC, and STA was made as follows. After melting P-188 in a water bath at 50 °C, it was stirred at 200 rpm for 20 min, followed by the addition of HPMC and STA with further 20 min of stirring, followed by the addition of DLZ and further stirring for 30 min. Size 1 hard gelatin capsules were used for the obtained liquefied dispersion. All preparations were left at a room temperature until the preparation solidified and then were kept in the fridge at 8 °C. 

For the first series, formulations of various ratios of DLZ to P-188 were made in the following ratio order: 1:9 (F1), 2:8 (F2), 3:7 (F3), 4:6 (F4), and 5:5 (F5) (*w*/*w*), respectively. All samples were prepared using the method described above for series 1. The total weight and percentages for each formulation is shown in Table 1. For the second series, formulations of various ratios of DLZ to a combination of P-188, HPMC, and STA were prepared following the method described previously for series 2. An increasing amount of STA was incorporated into the mixture in the following percentage order: 0% (F6), 2.5% (F7), 5% (F8), 7.5% (F9), 10% (F10) and 15% (F11). The total weights and percentages of all formulations are listed in Table 2. 

### 4.3. Drug Content Dermination

The content of DLZ in the polymer matrices of selected formulation (F8) and Tildiem^®^ was triturated separately in a mortar to a fine powder form. A known weight of powder mixture was then dispersed and dissolved in 100 mL of distilled water. The solution in the flask was filtered and the content of DLZ was determined after dilution, using spectrophotometry at a wavelength of 237 nm. The analysis was conducted in replicates of six and the average of drug content was then determined.

### 4.4. Dissolution Studies

For in vitro evaluation, dissolution experiments were run according to a previously reported method [57] in which the drug release was determined using 708-DS Dissolution Apparatus (Agilent Technologies, Santa Clara, CA, USA). The rotational speed of the paddle was set at 100 rpm and the volume of dissolution medium was filled to 900 mL with distilled water. The temperature was maintained at 37 °C. Samples were collected using a 1 mL pipette at the following time intervals: 0, 15, 30, 60, 90, 120, 180, 240, 300, 360, 390, 420, 480, 540, and 600 min. After sample collection, 1 mL of distilled water was added to maintain the volume of the dissolution medium. Similar dissolution studies evaluations on test formulation were performed but in various physiological simulated media, namely SGF, FaSSIF and FeSSIF, and they were prepared as described previously in the literature [58]. 

For measurement, a UV-spectrophotometer with the UV detector set at 237 nm was used. Each test was run in duplicate of six times and a standard calibration curve (0.6 to 23 µg/mL) was used for the drug analysis. The Korsmeyer-Peppas mathematical model was used to determine the kinetics of drug release according to the equation as described previously [31] (1):

Q = Kt*^n^* (1)

where Q represents the fraction of drug released at the specific time (t), K is a kinetic constant of drug release incorporating structural and geometrical characteristic of the matrix, and *n* is the drug release exponent. All obtained data were fitted to several kinetic drug release models as presented in Table 3.

### 4.5. Thermal Analysis

The thermal analysis of DLZ pure powder and test formulation (F8) was performed using differential scanning calorimetry (DSC) (Q100 OR3, New Castle, DE, USA). Powder samples of an average weight of 5.3 ± 0.3 mg were scanned between 100 °C and 250 °C at a rate of 10 °C/min. Nitrogen flow rate was set at 50 mL/min. Aluminium pans with lids were used for samples analysis. Each sample was run in triplicates.

### 4.6. In Vivo Animal Study 

The study was approved by Research Committee, Animal House, Ethical Approval No. RES-03-142.5. Twenty-four male rabbits (weight 4.2 ± 0.8 kg) were used. They were divided into two groups of 12 each. In the first phase of sampling, group 1 received one dose of Tildiem^®^ tablets (15 mg/kg) through oral gavage, while group 2 received one dose of polymer matrix test formulation (F8) (15 mg/kg) through oral gavage. Five mL of water was given to rabbits following the preparation administration. For sampling, 200 µL blood samples were taken from the ear vein and transferred into heparinized tubes at the following time intervals: 0, 0.25, 0.5, 0.75, 1, 1.5, 2, 3, 4, 6, 8, 10, 12, 14, 16, 24, 36, and 48 h. Collected samples were placed in Eppendorf and centrifuged at 15,000× *g* for 10 min. The obtained plasma samples were collected and stored at −21 °C prior to HPLC analysis.

### 4.7. HPLC Method for In Vivo Assessments and PK Parameters Determination 

The HPLC system (Shimadzu Corporation, Japan) used in this study was arranged with the same parts and reversed phase column (4.6 mm × 250 mm, 5 µm) as described in a previous study [46]. The mobile phase (MP) consisted of 0.2 M ammonium dihydrogen phosphate/isopropyl alcohol/acetonitrile/trimethylamine in a ratio of 54:44:1.5:0.5 (*v*/*v*). The pH was adjusted to 4.4 and the flow rate was set to 0.8 mL/min. The collected plasma samples (100 µL) were treated by adding a mixture of 400 µL of n-hexane and 2- propanol in a ratio of 95:5 (*v*/*v*). Treated samples were vortexed and centrifuged at 15,000× *g* for six min. Two layers were obtained, but only the top organic layer was used, evaporated, dried, mixed with 120 µL of MP, and injected immediately into the HPLC system. The method of non-compartmental model was used for analysis and in vivo plasma profile and PK parameters were determined as reported previously [59].

The HPLC assay was validated as described previously [60,61]. A duplicate of six batches of DLZ standard curves were prepared in water and in plasma with DLZ at final concentrations of 0.25, 0.5, 1, 2, 5, 10 and 20 µg/mL. The calibration standards were validated. Limit of quantitation (LOQ) and limit of detection (LOD) were determined. In addition, absolute recovery, method stability, intra-day, and inter-day accuracies and precisions were evaluated.

### 4.8. Statistical Analysis

The one-way analysis of variance method was used. The obtained results were considered statistically significant when *p <* 0.05.

## 5. Conclusions

In this study, the combination of P-188 with HPMC and STA showed excellent polymer matrix forming properties and successfully controlled the release rate of BCS class I drug DLZ for up to 10 h. Besides the simple way of preparation, this polymer matrix system was able to modify release of the drug as similar to Tildiem^®^, a commercially available tablet of DLZ. Therefore, the promising in vitro and in vivo results suggested that this particular dosage form could be an interesting alternative to traditional pharmaceutical systems for accommodating and controlling the release of other BCS class I drugs.

## Figures and Tables

**Table 1:** Controlled release polymer matrices containing various drug to polymer percentages.

Formulations	DLZ (%)	P-188t (%)	Total Weight (mg)
F1	10	90	600
F2	20	80	600
F3	30	70	600
F4	40	60	600
F5	50	50	600

**Table 2:** Controlled release polymer matrices containing various drug to polymer percentages with the addition of HPMC and STA.

Formulations	DLZ (%)	P-188 (%)	HPMC (%)	STA (%)	Total Weight (mg)
F6	20	30	50	0	300
F7	20	30	47.5	2.5	300
F8	20	30	45	5	300
F9	20	30	42.5	7.5	300
F10	20	30	40	10	300
F11	20	30	35	15	300

**Table 3:** Mechanism of DLZ release from controlled release polymer matrices using various kinetic release models represented by r^2^.

Model Name	r^2^ of F7	r^2^ of F8	r^2^ of F9	r^2^ of F10
Zero order model	0.9984	0.9816	0.9843	0.9857
First order model	0.7943	0.6778	0.6291	0.5769
Hixson-Crowell model	0.9751	0.9845	0.9877	0.9971
Higuchi model	0.9711	0.9874	0.9745	0.9686
Korsmeyer-Peppas model	0.4313	0.4409	0.3990	0.3829

**Table 4:** Extraction recovery, Intra-day and Inter-day accuracy and precision of the HPLC validation for the DLZ in plasma, (*n* = 6).

Concentration	Recovery		Intra-Day		Inter-Day	
(µg/mL)	Accuracy	Precision	Accuracy	Precision	Accuracy	Precision
	(%)	(C.V. %)	(%)	(C.V. %)	(%)	(C.V. %)
0.25	105.1	3.4	91.8	5.2	101.8	3.8
0.5	104.7	5.7	106.7	3.3	98.8	7.5
1	99.1	3.3	95.1	6.2	102.4	5.0
2	98.4	7.5	92.5	5.0	94.1	4.4
5	103.8	3.6	103.8	3.1	103.4	5.3
10	101.3	3.1	99.3	4.3	99.0	4.7
20	104.2	2.4	102.5	0.4	101.3	1.7

**Table 5:** Pharmacokinetic parameters of DLZ after in vivo oral administration of the commercial reference Tildiem^®^ (60 mg tablet) and test formulation (F8), data are mean ± SD, *n* = 12.

PK Parameters	Commercial Product (Tildiem^®^)	Test Formulation (F8)
C_max_ (ng/mL)	59.50 ± 11.7	66.81 ± 8.33
T_max_ (h)	8.00 ± 3.27	6.00 ± 2.42
MRT_0–∞_ (h)	50.5 ± 2.53	52.6 ± 3.71
AUC_0__–__∞_ (ng·h/mL)	1109.5 ± 352.7	1397.7 ± 352.9
AUC_0__–__t_ (ng·h/mL)	1065.1 ± 454.2	1300.7 ± 292.4
k_e_ (h^−1^)	0.074 ± 0.03	0.061 ± 0.04
t_½_ (h)	9.30 ± 4.25	11.4 ± 3.62

## Data Availability

Data is contained within the article.
